# Pitavastatin reduces intestinal fibrosis in chronic colitis and inhibits colon fibroblast activation by enhancing MMP-9 expression via the IGF-1/IGF-1R pathway

**DOI:** 10.1590/1414-431X2025e14540

**Published:** 2025-08-22

**Authors:** Mengran Zhang, Hongping Lu, Jun Cheng

**Affiliations:** 1 Department of Gastroenterology, Beijing Ditan Hospital, Capital Medical University, Beijing, China; 2 Hebei Utu Pharmaceutical Company Ltd., Shijiazhuang, Hebei Province, China

**Keywords:** Pitavastatin, Intestinal fibrosis, Inflammatory bowel disease, Fibroblast activation, IGF-1, MMP-9

## Abstract

Statins have been shown to have antifibrotic effects on various tissues and organs, but their ability to improve chronic colitis-associated intestinal fibrosis and their mechanisms of action remain unclear. The objective of this study was to investigate the role of pitavastatin in chronic colitis-associated intestinal fibrosis and its possible mechanisms. We established a mouse model of chronic colitis-associated intestinal fibrosis through repeated administration of dextran sodium sulfate (DSS) and treated the mice with pitavastatin. The severity of intestinal fibrosis, serum inflammatory factor levels, and expression levels of intestinal fibrosis-related genes in mice were assessed using pathological histological staining, immunohistochemical staining, reverse-transcription PCR, RNA sequencing, and enzyme-linked immunosorbent assay. *In vitro*, we treated a human colon fibroblast cell line (CCD-18Co) with or without transforming growth factor-β1 stimulation using pitavastatin. Western blot, Cell Counting Kit-8 assay, and Transwell assay were used to analyze the activation of colonic fibroblasts, protein expression levels of genes related to intestinal fibrosis, and cell proliferation and migration abilities. Pitavastatin significantly attenuated DSS-induced chronic colitis and intestinal fibrosis. *In vitro*, pitavastatin concentration-dependently inhibited the activation of CCD-18Co cells, significantly reduced the expression levels of the intestinal fibrosis-related proteins Col1A1, IGF-1, IGF-1R, MMP-3, and TIMP-1, and significantly inhibited cell proliferation and migration while markedly increasing MMP-9 protein expression. Additionally, after silencing the IGF-1 and IGF-1R genes in CCD-18Co cells, the promotion of MMP-9 expression by pitavastatin was significantly inhibited. These findings suggest that pitavastatin may be a promising antifibrotic drug for future treatment of intestinal fibrosis.

## Introduction

Ulcerative colitis (UC) is a non-specific inflammatory disease of the intestine characterized by chronic inflammation and ulceration of unknown cause. Long-term recurrent intestinal inflammation leads to an imbalance between the synthesis and degradation of the extracellular matrix (ECM), ultimately causing intestinal fibrosis (1), which seriously affects intestinal function and therapeutic efficacy. Historically, UC-associated mucosal lesions were thought to be superficial, and intestinal fibrosis was generally believed to be limited to the submucosal layer and to have a low incidence. However, recent studies have shown that intestinal fibrosis occurs in both the acute and chronic phases of UC (2) and that almost all patients have some degree of intestinal fibrosis (1,3,4). The exact mechanism of its occurrence remains incompletely understood.

Despite the increased use of anti-inflammatory and biologic therapies to induce remission and temporarily improve obstructive symptoms, evidence suggests that anti-inflammatory drugs neither prevent nor attenuate intestinal fibrosis and that fibrosis continues to progress even with well-controlled intestinal inflammation (5). Additionally, 40% of patients with Crohn's disease still undergo surgery or dilation within 12 months (6) and 70-90% experience recurrence of intestinal strictures within 1 year of surgery (7). This indicates that the development of intestinal fibrosis may be an independent, ongoing, and self-regulated process, with cellular or molecular factors contributing independently of inflammation. This situation creates a unique heterogeneous condition in the intestinal tract. The precise cause is unknown, making intestinal fibrosis more complex than fibrosis in other organs. Effective preventive and antifibrotic treatments are lacking, highlighting the need for drugs that can reverse or prevent fibrosis.

Recent studies have shown that the activation of intestinal fibroblasts is a key step driving intestinal fibrosis (8). During the initial stage of inflammation, fibroblasts proliferate and activate in response to various cytokines and inflammatory mediators. Activated fibroblasts secrete large amounts of cytokines, causing excessive deposition of submucosal ECM and leading to intestinal fibrosis (9).

Pitavastatin (PV) is a commonly used oral lipid-lowering drug that inhibits hepatic cholesterol synthesis. Preclinical and clinical studies have shown that statins have antifibrotic activity. Atorvastatin has been confirmed to reduce fibrosis in a mouse model of renal protection (10). Simvastatin was shown to inhibit the development of cardiovascular fibrosis in an angiotensin II-induced mouse model (11). A case-control study involving 42,272 patients showed that statin use was associated with a lower risk of idiopathic pulmonary fibrosis and higher overall survival (12). Elbaset et al. (13) reported that pitavastatin prevented hepatic fibrosis in rats by attenuating oxidative stress and inhibiting the NF-κB and PI3K/Akt signaling pathways. Some recent studies have also revealed that statins exert antifibrotic effects in the gut (14,15). Therefore, statins may have therapeutic value for chronic enteritis-associated intestinal fibrosis, although further investigation is required to elucidate the underlying mechanisms.

In the present study, we investigated the inhibitory effect of pitavastatin in a dextran sodium sulfate (DSS)-induced chronic enteritis model and on the activation of human colonic fibroblasts. We evaluated the ability of pitavastatin to suppress fibroblast activation, proliferation, migration, and secretion of inflammatory cytokines, as well as its effects on ECM synthesis. The underlying mechanism may involve promotion of matrix metalloproteinase-9 (MMP-9) expression through the insulin-like growth factor 1 (IGF-1)/insulin-like growth factor 1 receptor (IGF-1R) pathway.

## Material and Methods

### Reagents and antibodies

Dextran sulfate sodium (DSS, 36-50 kDa) was obtained from MP Biomedicals (USA). Pitavastatin (HMGCR inhibitor) was purchased from MCE (USA). Recombinant human TGF-β1 (rhTGF-β1) was obtained from R&D Systems (USA). Primary antibodies against IGF-1 (ab223567), IGF-1R (ab131476), TGF-β1 (ab215715), α-SMA (ab124964), COL1A1 (ab138492), MMP-3 (ab52915), MMP-9 (ab76003), and TIMP-1 (ab211926) were purchased from Abcam (USA). Primary antibodies to COL3A1 (#30565) and GAPDH (#5174) were purchased from Cell Signaling Technology (USA). The short interfering RNA (siRNA) oligonucleotides against human IGF-1R were purchased from GenePharma (China).

### Animal experiments and drug treatment

**Figure 1:**
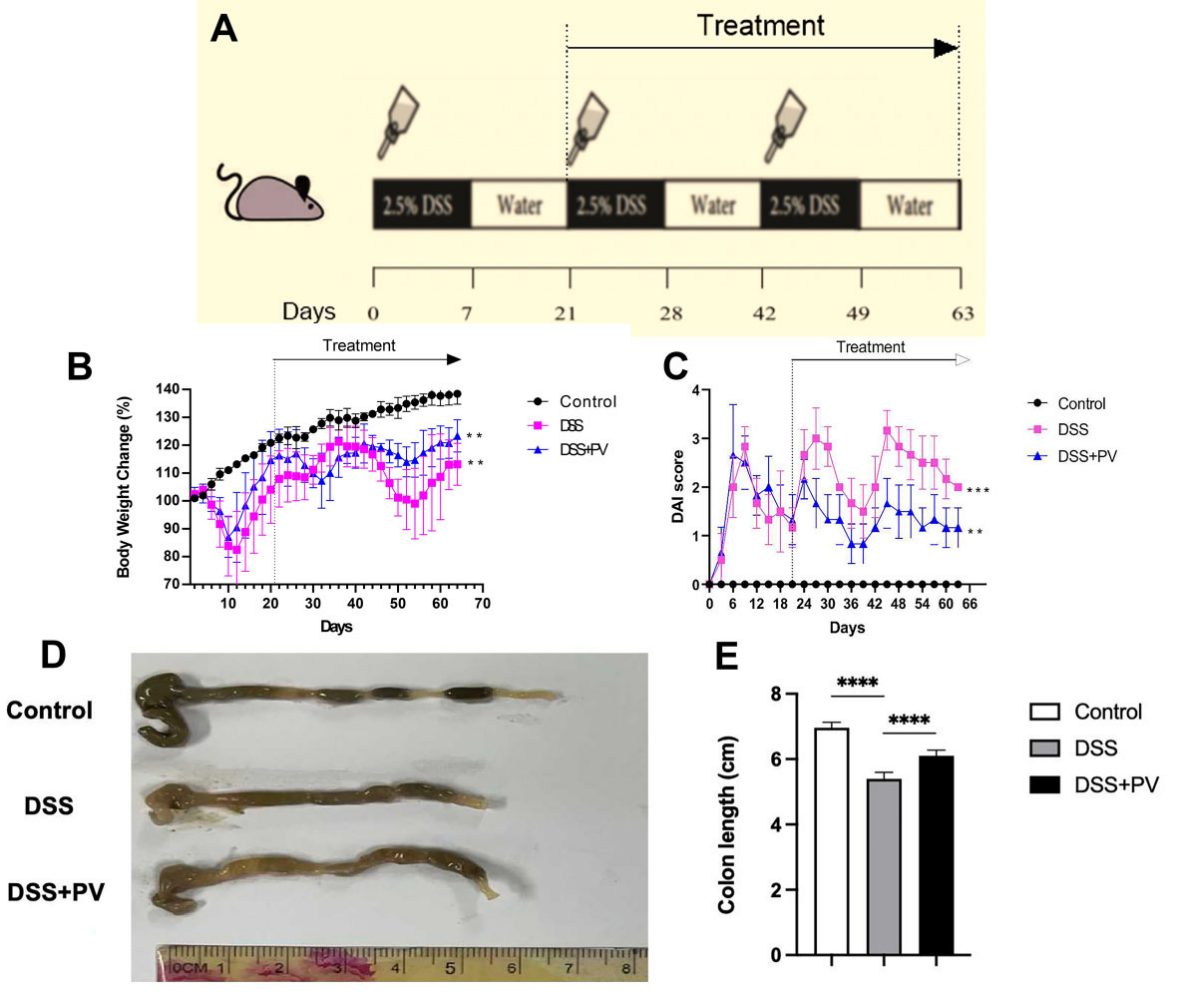
Pitavastatin (PV) treatment reduced intestinal fibrosis in the dextran sodium sulfate (DSS) mouse model. **A**, Mouse experimental protocol of DSS colitis (treatment from day 21 to day 63). **B**, Body weight change was recorded during the experiment. **C**, Disease activity index (DAI) scores were recorded at certain time points. **D**, Representative colons from different groups. **E**, Colon length in different groups. Data are reported as means±SD. **P<0.01, ***P<0.001 weight changes and DAI scores in the DSS group were statistically significant compared with the control group; weight changes and DAI scores in the DSS+PV group were statistically significant compared with the DSS group. ****P<0.0001 *vs* the DSS group (ANOVA).

Male C57BL/6 mice (18-20 g, 6 weeks old) were procured from the Institute of Laboratory Animals Science (CAMS & PUMC, China) and housed at a specific pathogen-free (SPF) care facility with a 12-h light/dark cycle and free access to water and food for seven days prior to the beginning of experiments. Then, the mice were randomly divided into three groups: control group (n=10), DSS group (n=10), and DSS+Pitavastatin group (n=10). Experimental chronic inflammation and fibrosis were induced by three consecutive cycles of 2.0% DSS in drinking water for 7 days followed by 2 weeks recovery with water without DSS according to a previous study (16). The timelines of the experiment are shown in Figure 1A. In brief, the control group only received water during the experiment. For therapeutic purposes, after one cycle of DSS (from day 21), the mice in the DSS+Pitavastatin group were administered pitavastatin (8 mg/kg, once/day) intragastrically. The control and DSS groups were treated with the same dose of saline. The body weight change in each group was measured once a day, and the disease activity index (DAI) was assessed at a specific time during the treatment period. DAI scores were blindly evaluated as described previously (17). The DAI scoring system consists of three parts: weight loss, stool consistency, and blood in feces (Supplementary Table S1). DAI total score range is 0-12, which is calculated with the formula: DAI = (Weight Loss Score + Stool Consistency Score + Blood in Feces Score) / 3. A score of 0 means that the three indicators are normal, and the closer the score is to 4, the more serious the inflammation. All mice were euthanized at the end of the experiment, and their blood samples were collected from the orbit. The colons were quickly removed, and their lengths were recorded. Subsequently, a portion of the colon tissue was formalin-fixed and then paraffin-embedded. The remaining colon tissue was kept at −80°C.

### Histopathological evaluation

For the histopathological assay, the distal colon sections were stained with hematoxylin and eosin (HE), Sirius red, and Masson staining. Histologic alterations were measured in HE sections. Collagen deposition was assessed with Sirius red and Masson staining (18). The histopathological evaluation of the colon was performed according to previously described methods (17,19) and in a blind manner. Inflammation level and epithelium were scored and scores varied from 0 to 4.

### Cytokine quantification

Serum was obtained from whole blood samples of mice by centrifugation (1200 *g*, 15 min, 4°C). The concentrations of interleukin (IL)-6, IL-10, tumor necrosis factor-α (TNF-α), and interferon-γ (IFN-γ) were determined by respective enzyme-linked immunosorbent assay (ELISA) kits based on the manufacturer's protocols.

### RNA isolation and quantitative real-time PCR (real-time qPCR)

Total RNA was extracted from colon tissues of mice (Total RNA Kit, USA) and reverse transcribed into a single-strand cDNA (Prime Script RT Reagent Kit, TaKaRa, China). Then, real-time PCR was performed in triplicate and normalized to β-actin using SYBR Green qPCR Master Mix (Applied Biosystems, UK) on an ABI 7500 System (Applied Biosystems, USA). The ratio of expression of the target gene to the reference gene was estimated according to the 2^-ΔΔct^ method. The primer sequences to detect COL1A1, COL3A1, MMP-3, TGF-β1, IGF-1, TNF-α, and TIMP-1 are shown in Table 1.

**Table 1:** Primers used in the qPCR assay.

Gene	Primers	Sequences (5′-3′)
*COL1A1*	Forward	TTCTCGGCAAAGACGGAC
	Reverse	CGGCCACCATCTTGAGACTT
*COL3A1*	Forward	AAGGCTGCAAGATGGATGCT
	Reverse	GTGCTTACGTGGGACAGTCA
*MMP-3*	Forward	GGCGCAAATCTCTCAGGACT
	Reverse	TCTTCTTCACGGTTGCAGGG
*TGF-β1*	Forward	ACTGGAGTTGTACGGCAGTG
	Reverse	GGGGCTGATCCCGTTGATTT
*IGF-1*	Forward	GCCTCATTATCCCTGCCCAC
	Reverse	CGCCAGGTAGAAGAGGTGTG
*TNF-α*	Forward	TAGCCCACGTCGTAGCAAAC
	Reverse	GCAGCCTTGTCCCTTGAAGA
*TIMP-1*	Forward	GAGACCACCTTATACCAGCGT
	Reverse	TACGCCAGGGAACCAAGAAG
*β-actin*	Forward	GTACTCTGTGTGGATCGGTGG
	Reverse	GCAGCTCAGTAACAGTCCG

### Protein extraction and western blot analysis

Cell lysates were prepared with RIPA buffer, protease, and phosphatase inhibitors (Fudebio, China). The protein concentration was determined by the Pierce BCA assay (Thermo Scientific, USA). After denaturation, the 12% SDS-PAGE was used to separate proteins. Then, the proteins were transferred onto polyvinylidene difluoride (PVDF) membranes (Millipore, USA). After being blocked with 5% skim milk, the membranes were incubated with primary antibodies at 4°C overnight and secondary antibodies for 1 h at 37°C. Signals detected using the ECL kit (K-12094-D50, Advansta, USA) were quantified using ImageJ software (NIH, USA) and normalized to GAPDH levels.

### Cell culture, transfection, and treatment

CCD-18Co cells were kindly gifted by Prof. Wei Zhifeng (China Pharmaceutical University, China). CCD-18Co cells were maintained in Eagle's Minimum Essential Medium (EMEM, ATCC) supplemented with 10% fetal bovine serum (Gibco, USA) at 37°C and 90% humidity in a 5% CO_2_ incubator. Cell transfection was performed with the PolyPlus-transfection reagent (PolyPlus-Transfection SA, USA) according to the manufacturer's protocol. After starving (0% serum) for 24 h, the cells were treated with rhTGF-β1 (2.5 ng/mL) for 24 h and then treated with different concentrations of pitavastatin (100, 200, or 300 nM) for 24 h. Untreated cells and cells treated with rhTGF-β1 alone were regarded as controls.

### Proliferation assay

CCD-18Co cells were seeded onto a 96-well plate and deprived of serum for 24 h before the experiment. Cell Counting Kit-8 (CCK8) solution (DOJINDO, Japan) was added to each well and incubated at 37°C for 1 h. The absorption was detected at 450 nm.

### Migration assay

Transwell (8-μm pore size, Millipore) tests were used for CCD-18Co migration assays. Starving cells were harvested and seeded onto the upper chambers with serum-free medium supplemented with rhTGF-β1 or pitavastatin. The lower chambers contained EMEM with 20% FBS. Twenty-four hours later, cells that migrated to the lower surface were fixed and dyed with crystal violet solution.

### Immunohistochemistry (IHC)

IHC was conducted according to the instructions of the Universal Two-step Detection kit (Zhongshan Golden Bridge, China). Colon sections were incubated with primary antibody overnight at 4°C, and HRP-conjugated anti-rabbit antibody was incubated at room temperature for 40 min and the blots were then developed with the DAB kit (Zhongshan Golden Bridge). Immunohistochemical staining results were assessed by IRS semi-quantitative analysis by combining staining intensity and the proportion of positive cells. Staining intensity scoring criteria were as follows: 0 (none), 1 (mild), 2 (moderate), and 3 (strong). The percentage of positive cells was scored on the following scale: 0 (none), 1 (1-25% positive cells), 2 (26-50% positive cells), 3 (51-75% positive cells), and 4 (76-100% positive cells). The final composite score was the product of the staining intensity score and the positive cell percentage score and was categorized as negative (0), weakly positive (1-4), moderately positive (5-8), and strongly positive (9-12).

### Gene cluster analysis

The gene cluster analysis of the colon tissue in DSS-induced UC mice was carried out by the Beijing Center for Physical and Chemical Analysis (China).

### Statistical analysis

Student's *t*-test was used for comparisons of two groups, and one-way ANOVA was used for multi-group comparisons. Data were obtained from at least three independent trials. Data are reported as means±SD. The statistical analysis was performed using SPSS 26.0, with differences of P<0.05 considered statistically significant.

## Results

### Pitavastatin attenuated chronic colitis-related intestinal fibrosis induced by DSS

**Figure 2:**
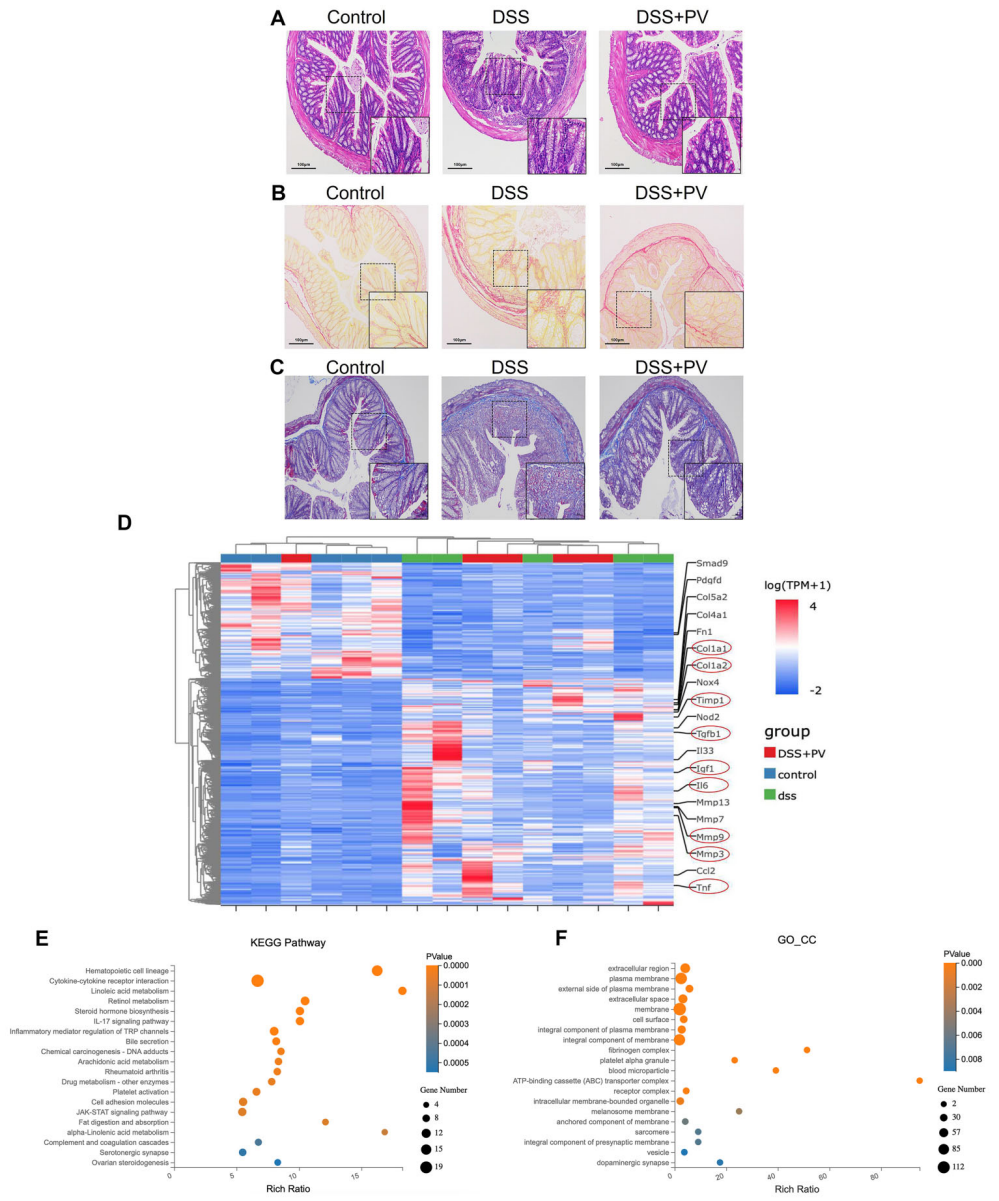
The relieving effect of pitavastatin (PV) in the histological evaluation and fibrosis-related gene expressions. **A**-**C**, Colon tissues were studied by (**A**) hematoxylin and eosin staining, (**B**) Sirius Red staining, and (**C**) Masson staining. Scale bars 100 μm. **D**, Clustering heatmap of differentially expressed genes (DEGs) in the colon tissues of different groups of mice. **E** and **F**, Functional enrichment analysis of DEGs was performed using Kyoto encyclopedia of genes and genomes (KEGG) (**E**) and Gene Ontology (GO) (**F**) in the dextran sodium sulfate (DSS) and DSS+PV groups.

In this study, we analyzed the potential therapeutic effects of pitavastatin in a DSS-induced chronic enteritis model. After DSS intake, mice exhibited significant weight loss and elevated DAI scores, but pitavastatin treatment dramatically ameliorated these changes compared with the DSS group (Figure 1B and C). DSS also caused colonic shortening, which was significantly improved by pitavastatin treatment (Figure 1D and E). Histological examination of colon slices from the DSS group using HE staining showed massive inflammatory cell infiltration, disturbed crypt structure, and ulcer formation. Pitavastatin treatment attenuated this histopathological damage (Figure 2A). To assess colonic fibrosis, we performed Sirius red and Masson staining, which showed strong collagen fiber deposition in the DSS group. Pitavastatin treatment significantly reduced this deposition (Figure 2B and C).

**Figure 3:**
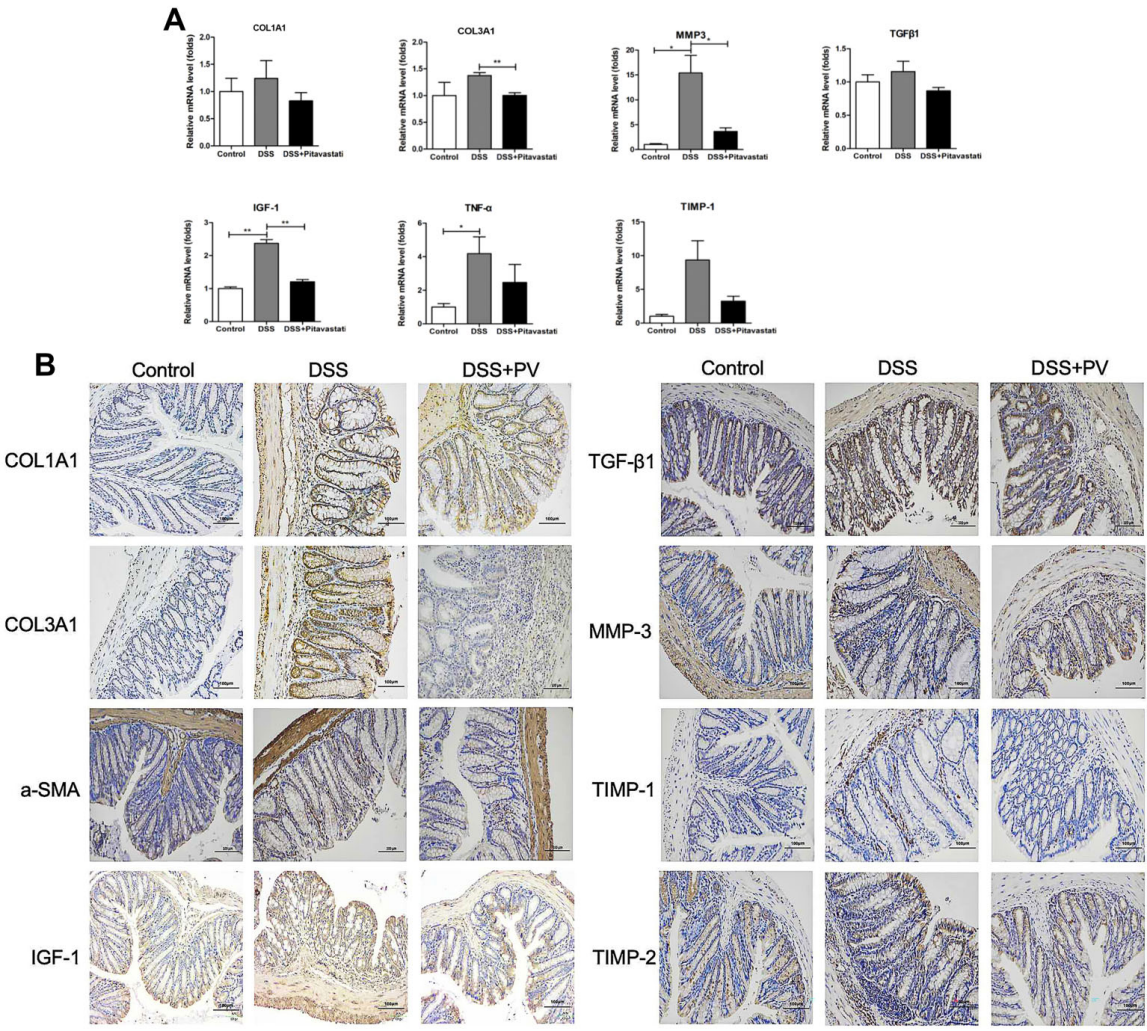
The relieving effect of pitavastatin (PV) in the histological evaluation and fibrosis-related gene expressions. **A**, mRNA expression of fibrotic markers was analyzed via real-time PCR of colon tissues. Data are reported as means±SD. *P<0.05; **P<0.01. Quantitative results were analyzed by one-way ANOVA. **B**, Representative micrographs of immunohistochemical staining for Col1A1, Col3A1, a-SMA, IGF-1, TGF-β1, MMP-3, TIMP-1, and TIMP-2 in colon specimens. Scale bars, 100 μm.

The activation of colonic fibroblasts is a crucial initiating event in the development of fibrosis, with α-smooth muscle actin (α-SMA) serving as a marker of fibroblast activation. This activation leads to increased ECM synthesis and cell proliferation, ultimately causing fibrosis. We determined whether pitavastatin can inhibit fibroblast activation, ECM synthesis, and fibrotic protein expression (Figure 2D-F). RNA sequencing of colonic tissues from the DSS group and DSS+Pitavastatin group in mice identified 355 differentially expressed genes. KEGG (Kyoto Encyclopedia of Genes and Genomes) enrichment analysis of these genes revealed a series of significantly enriched pathways (Figure 2E). Results showed that pitavastatin may exert its anti-fibrotic effects by modulating cytokine-receptor interactions, the JAK-STAT signaling pathway, cell adhesion molecules, the IL-17 signaling pathway, and the complement and coagulation cascades, thereby inhibiting fibroblast activation and inflammation. As shown in Figure 2F, the GO-CC (Gene Ontology Cellular Component) enrichment analysis of these differentially expressed genes revealed that pitavastatin may influence cellular structure and function during intestinal fibrosis by regulating ECM synthesis (e.g., collagen, fibronectin), secretion of extracellular molecules (e.g., cytokines, growth factors), expression of fibrinogen, as well as receptors and adhesion molecules on the cell membrane, and transmembrane proteins (e.g., TGF-β receptors, TRP channels). As shown in Figure 3A, RT-qPCR revealed elevated mRNA expression of *COL1A1*, *COL3A1*, *MMP-3*, *TGF-β1*, *IGF-1*, *TNF-α*, and *TIMP-1* in DSS-induced intestinal fibrosis compared with controls. Pitavastatin treatment markedly decreased the mRNA expression of *COL3A1*, *IGF-1*, and *MMP-3*.

To investigate the underlying mechanisms of the antifibrotic effects of pitavastatin, we evaluated its impact on IGF-1 expression and ECM synthesis, considering that IGF-1 is a key regulator of the formation of intestinal fibrosis. Immunohistochemical staining showed increased expression of Col1A1, COL3A1, IGF-1, and α-SMA in DSS-treated mice compared with controls, and the expression of each decreased after pitavastatin treatment (Figure 3B). These findings were consistent with the RT-qPCR results.

### Pitavastatin decreased the production of proinflammatory cytokines in colon tissue with DSS-induced colitis and intestinal fibrosis

Inflammatory cytokines play a crucial role in the pathogenesis of colitis and intestinal fibrosis. When the colonic mucosal epithelium is disrupted by DSS, it triggers a cascade of inflammatory responses in the colonic tissues, contributing to the formation of intestinal fibrosis.

**Figure 4:**
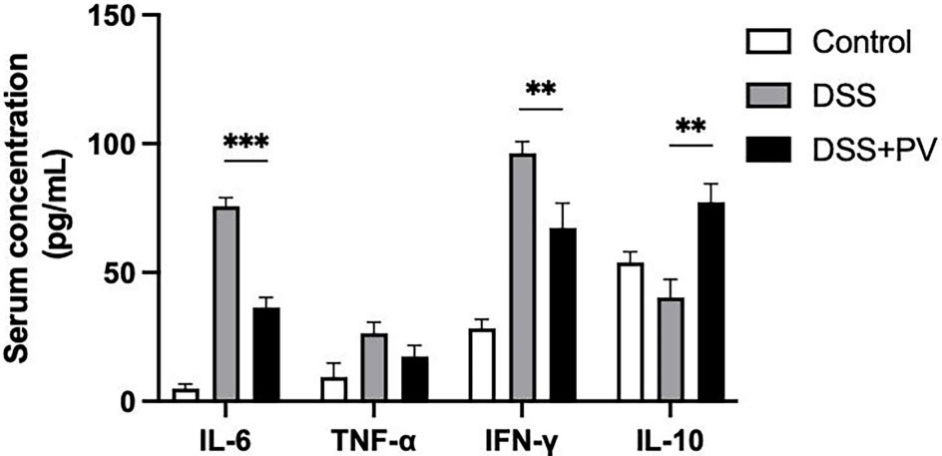
Pitavastatin (PV) regulated the expression of serum inflammatory cytokines interleukin (IL)-6, tumor necrosis factor (TNF)-α, interferon (IFN)-γ, and IL-10 in the dextran sodium sulfate (DSS)-induced chronic colitis and intestinal fibrosis mouse model. Data are reported as means±SD. **P<0.01; ***P<0.001 (ANOVA).

As shown in Figure 4, proinflammatory cytokines (IL-6, TNF-α, and IFN-γ) significantly increased in DSS-treated mice relative to the control group, but they were suppressed after pitavastatin treatment. By contrast, expression of the anti-inflammatory cytokine IL-10 significantly increased with pitavastatin treatment.

### Pitavastatin inhibited transforming growth factor-β1 (TGF-β1)-induced activation and ECM synthesis of human colon fibroblasts *in vitro*

**Figure 5:**
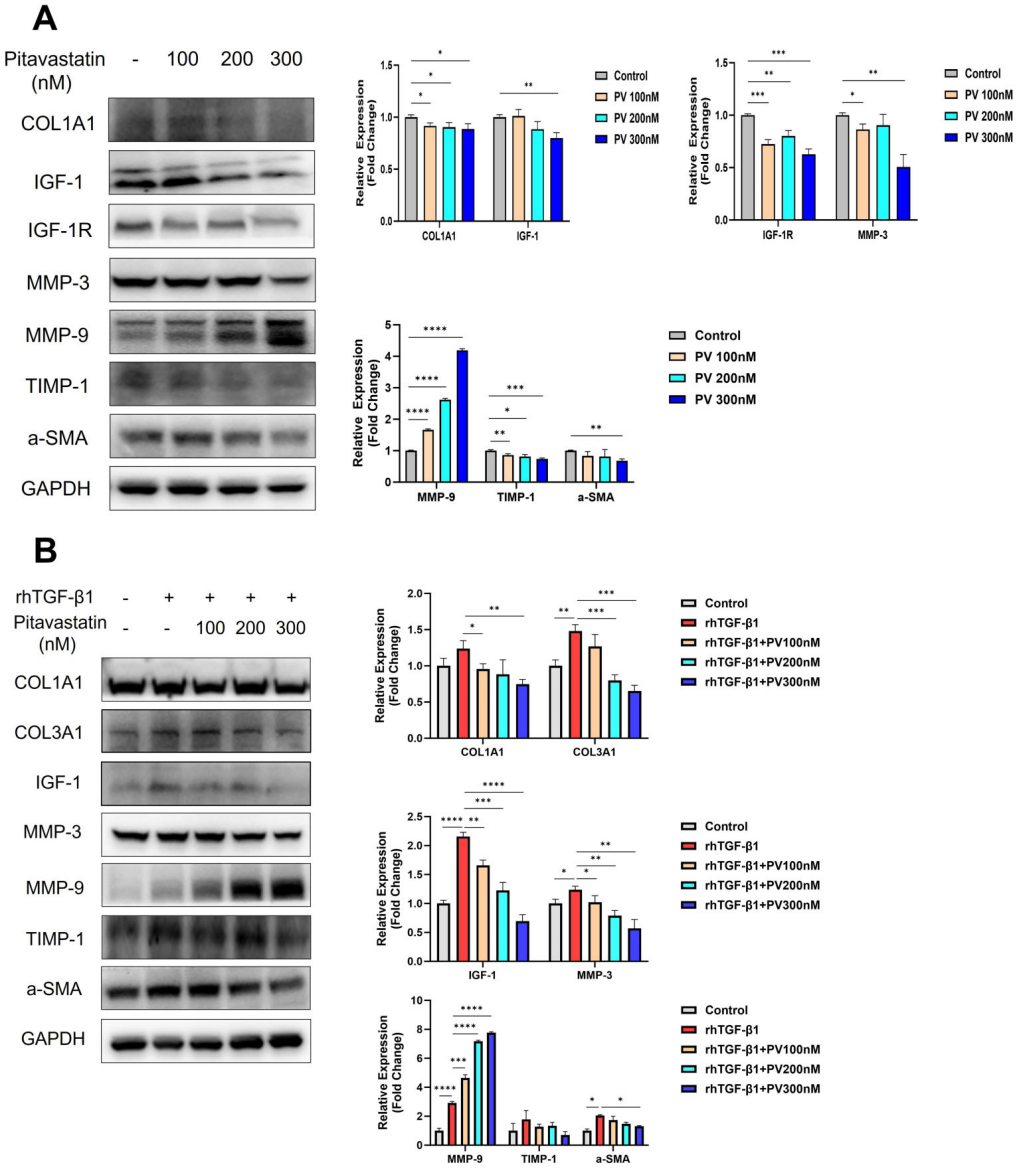
Pitavastatin (PV) dose-dependently attenuated basal and tumor growth factor (TGF)-β1-induced human colon fibroblasts activation and fibrosis-related protein expression *in vitro*. **A**, Protein expressions of Col1A1, IGF-1, IGF-1R, MMP-3, MMP-9, TIMP-1, and α-SMA in CCD-18Co cells after PV treatment. Relative densitometry values are indicated beside blots as ratios relative to GAPDH. **B**, Protein expressions of Col1A1, Col3A1, IGF-1, MMP-3, MMP-9, TIMP-1, and α-SMA. Relative densitometry values are indicated beside blots as ratios relative to GAPDH. Data are reported as means±SD. *P<0.05; **P<0.01; ***P<0.001; ****P<0.0001 (ANOVA).

CCD-18Co cells, a human colon fibroblast cell line, are considered the main source of ECM in intestinal fibrosis. Therefore, we investigated the attenuating effect of pitavastatin on the production and degradation of ECM-related proteins in TGF-β1-treated CCD-18Co cells. To assess the dose-dependent bioactivity of pitavastatin and select the optimal concentration, experiments were performed at concentrations of 100, 200, and 300 nM. Pitavastatin inhibited the activation of CCD-18Co cells in a concentration-dependent manner. It dose-dependently reduced the basal protein expression levels of α-SMA, Col1A1, IGF-1, IGF-1R, MMP-3, and TIMP-1 while significantly increasing the expression of MMP-9 (Figure 5A), a protein related to fibroblast activation and ECM deposition.

The CCD-18Co cells were activated by TGF-β1 (2.5 ng/mL) for 24 h and then treated with different concentrations of pitavastatin for a further 24 h. We found that pitavastatin at 300 nM markedly promoted the restoration of TGF-β1-activated myofibroblasts to the quiescent state and diminished the upregulation of Col1A1 and COL3A1 induced by TGF-β1. This effect was achieved by increasing MMP-9 while inhibiting IGF-1, MMP-3, and TIMP-1, as shown by the protein levels (Figure 5B).

### Pitavastatin alleviated TGF-β1-induced proliferation and migration of human colonic fibroblasts *in vitro*

**Figure 6:**
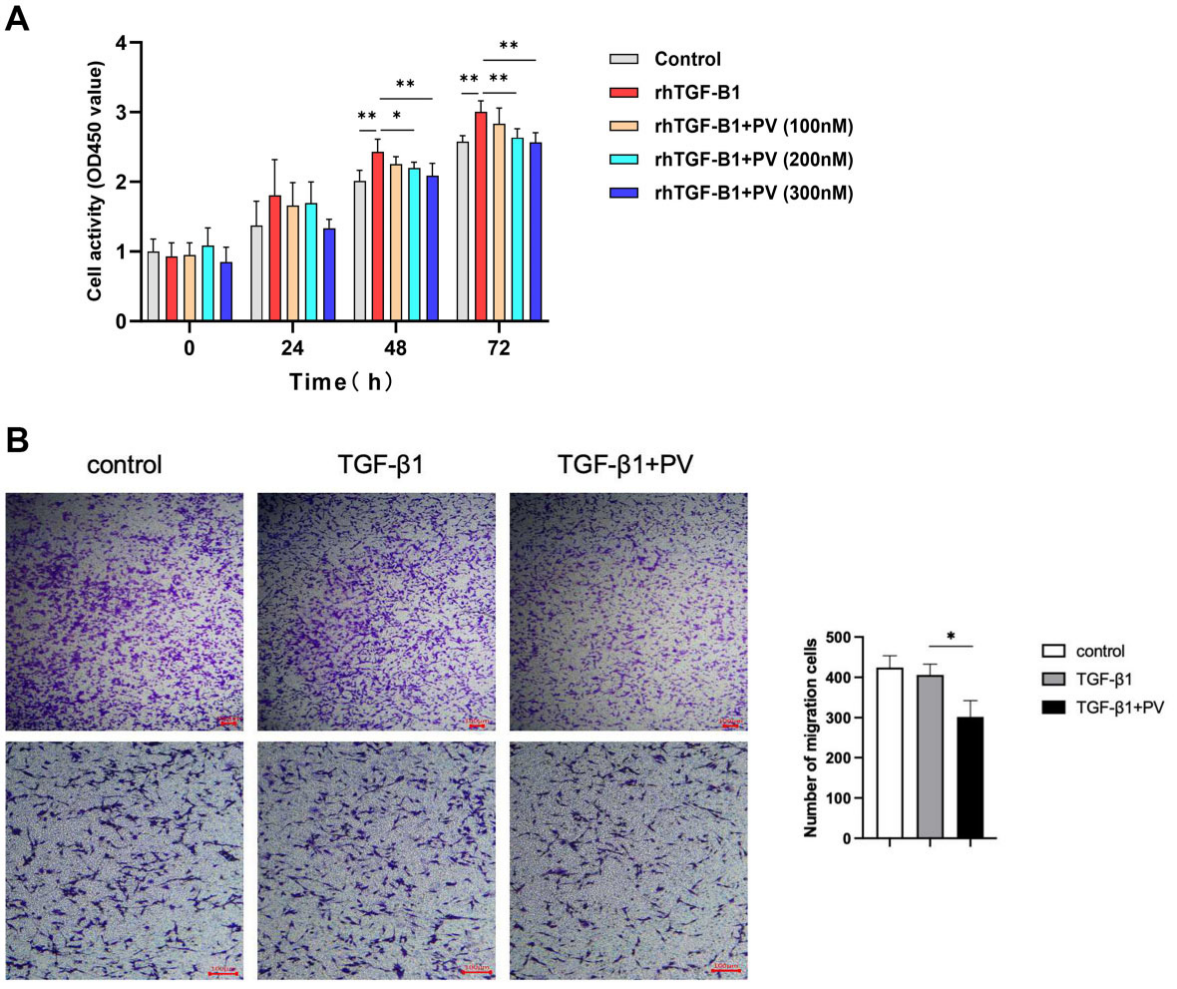
Pitavastatin (PV) dose-dependently alleviated tumor growth factor (TGF)-β1-induced proliferation and inhibited migration in cultured human colon fibroblasts. **A**, Proliferation of human colon fibroblasts treated with or without PV was tested by CCK-8 assay incorporation in response to 2.5 ng/mL rhTGF-β1. **B**, Cell motility was monitored by Transwell assay. Representative microscopy images and quantifications of migrating cells are shown. Scale bars 100 μm. Data are reported as means±SD. *P<0.05; **P<0.01 (ANOVA).

Proliferation is an essential behavior of myofibroblasts in fibrogenesis. A CCK-8 assay was performed to further investigate the potential antifibrotic mechanism of pitavastatin. The assay revealed that the proliferative response was significantly enhanced in TGF-β1-treated CCD-18Co cells, but this effect was alleviated after pitavastatin treatment at both lower and higher dosages at 48 and 72 h (Figure 6A), with the higher dosage showing a significant effect. Additionally, a Transwell assay demonstrated that TGF-β1 stimulation did not affect cell migration capacity, but pitavastatin at 300 nM markedly inhibited fibroblast migration (Figure 6B).

These results suggest that pitavastatin regulates the activation, ECM synthesis, proliferation, and migration of colonic myofibroblasts *in vitro*.

### Pitavastatin attenuated fibrosis by increasing MMP-9 expression through IGF-1/IGF-1R in cultured human colonic fibroblasts

**Figure 7:**
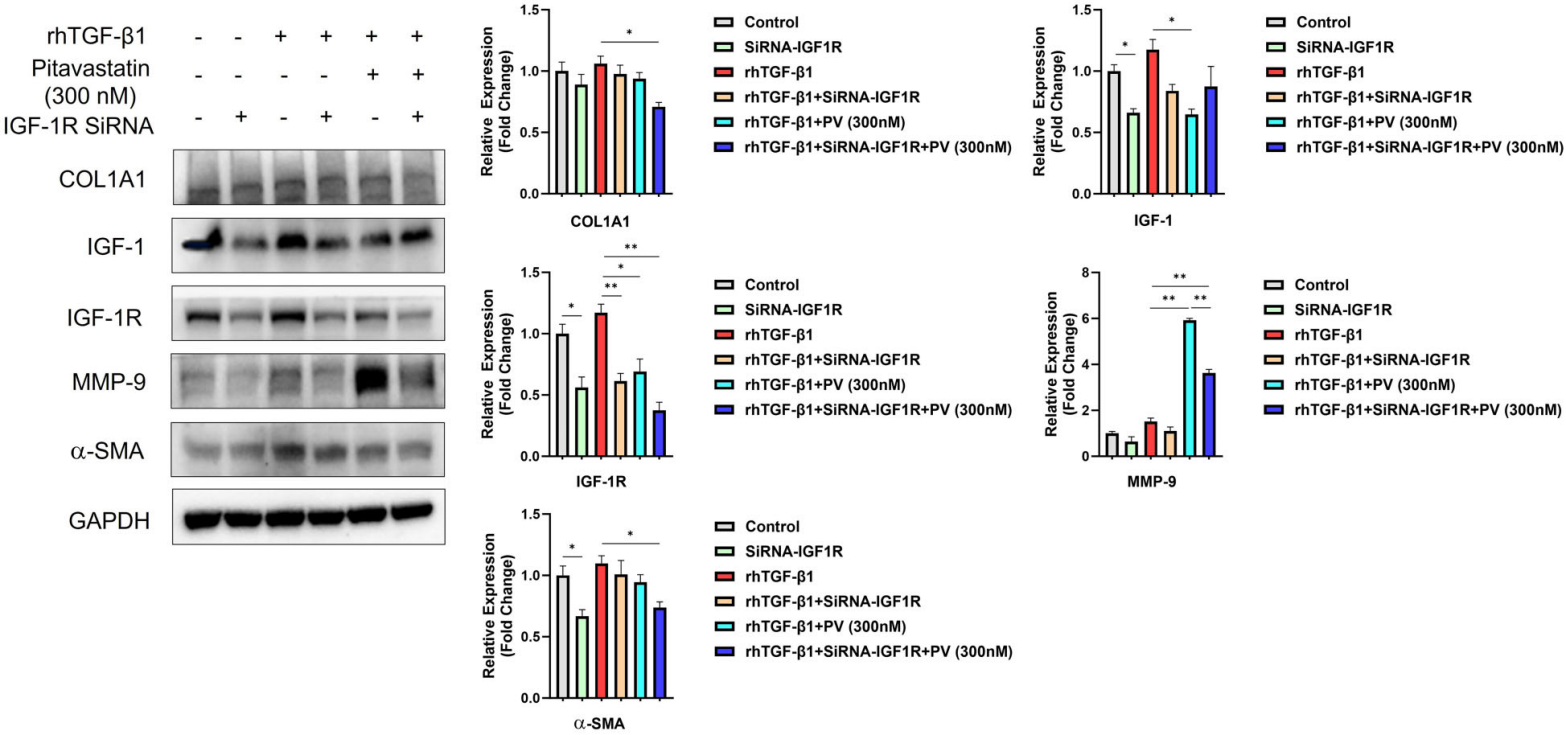
Pitavastatin (PV) attenuated fibrosis by increasing MMP-9 expression through IGF-1/IGF-1R in cultured fibroblasts. After being infected with scrambled or IGF-1R-specific siRNA, tumor growth factor (TGF)-β1-induced CCD-18Co cells were further treated with PV. Protein expression of Col1A1, IGF-1, IGF-1R, MMP-9, and α-SMA are shown by western blot analysis. Relative density of each protein to GAPDH are shown. Data are reported as means±SD. *P<0.05; **P<0.01 (ANOVA).

To further evaluate the effect of pitavastatin on the IGF-1/IGF-1R pathway, we investigated the impact of IGF-1R knockdown on fibroblast activation and collagen production using siRNA in TGF-β1-induced CCD-18Co cells. Western blot analysis demonstrated that IGF-1 and IGF-1R protein levels were significantly lower in knockdown cells than in cells treated with scrambled siRNA. Furthermore, the expression of α-SMA, Col1A1, and MMP-9 was decreased by IGF-1R knockdown both in the presence and absence of TGF-β1 stimulation. Compared with IGF-1R suppression in the presence of TGF-β1 stimulation, pitavastatin also significantly reduced α-SMA and Col1A1 protein expression but notably increased MMP-9 expression. When combined with siRNA IGF-1R, pitavastatin further down-regulated the expression of α-SMA and Col1A1, although the enhancement of MMP-9 expression was significantly attenuated (Figure 7).

## Discussion

Intestinal fibrosis is responsible for most of the long-term complications and surgical interventions in patients with inflammatory bowel disease. Despite improved insights into the pathogenesis of intestinal fibrosis (10,20), effective antifibrotic therapies remain scarce. Potential adverse effects limit the use of current therapeutic agents (21). Emerging evidence suggests that anti-inflammatory drugs do not prevent or attenuate intestinal fibrosis and that fibrosis continues to progress despite well-controlled intestinal inflammation, indicating that mechanisms independent of inflammation may control intestinal fibrosis (20). Thus, the development of new drugs that can blunt or reverse fibrosis is of paramount importance. Alongside developing new drugs, repurposing existing drugs may be an effective strategy to identify antifibrotic compounds, saving time and costs and providing advantages over *de novo* drug discovery (22). Increasing evidence has demonstrated that statins have pharmacological activity against fibrosis in addition to their common uses for treating dyslipidemia and cardiovascular disease. Moreover, statins have been found to inhibit gut fibrosis in recent studies (15,23), although the mechanism is unclear.

In this study, we found that pitavastatin attenuated DSS-induced chronic colitis and intestinal fibrosis. The antifibrosis mechanisms of pitavastatin may be attributed to its suppression of intestinal fibroblast activation, proliferation, and migration and its reduction of intestinal inflammatory factors. Pitavastatin decreased collagen synthesis by increasing MMP-9 expression via the IGF-1/IGF-1R pathway.

Recent studies have shown that the activation of fibroblasts is a key step driving the development of intestinal fibrosis (8). Activated fibroblasts undergo phenotypic and functional changes, transforming into myofibroblasts with significantly enhanced ECM synthesis capacity (9). In this study, we observed marked thickening of the submucosa and significant accumulation of collagen in mice with DSS-induced intestinal fibrosis. Additionally, fibrotic tissues in the colon were characterized by overexpression of TGF-β1/IGF-1 signaling proteins and high expression of α-SMA, a marker of fibroblast activation, and TIMP-1 (Figure 3B).

IGF-1 is a crucial regulator of intestinal smooth muscle proliferation and collagen accumulation. It has been demonstrated to facilitate mucosal proliferation, repair damage, and prevent apoptosis, thus inhibiting intestinal fibrosis and stricture formation (24). A recent study showed that heterozygous mice with an *IGF-1* gene deletion exhibited a reduction in intestinal fibrosis in a trinitrobenzene sulfonic acid-induced colitis model (25). Other studies have shown that elevated circulating or local IGF-1 expression may exacerbate intestinal fibrosis during inflammation or injury (26,27), prompting considerable interest in IGF-1 as an endogenous mediator or potential therapeutic target. In this study, we observed a significant elevation in both the mRNA and protein expression levels of IGF-1 in colonic tissues of mice with DSS-induced intestinal fibrosis, consistent with previous findings. Oral administration of pitavastatin significantly reduced IGF-1 expression levels. IGF-1 plays a double-edged role in mucosal healing and repair, but its regulatory mechanism in intestinal fibrosis remains unclear and needs further research.

Given previous studies demonstrating the antifibrotic effects of statins in organs such as the intestine, lungs, and kidneys, we believe that pitavastatin may exert a protective effect on chronic colitis and the associated intestinal fibrosis. In this study, we showed that pitavastatin relieved established intestinal fibrosis, alleviated intestinal inflammation, and improved histological damage in a mouse model of DSS-induced chronic colitis. Pitavastatin is used in patients with hypercholesterolemia at dosages of 2-4 mg/day. Because of differences in the body surface area and other species-related factors between humans and mice, pitavastatin has been administered in mice at doses of 1-15 mg/kg according to the literature. Therefore, we chose a dosage of 8 mg/kg per day, which was confirmed to be effective. These findings suggest that pitavastatin at moderate doses might inhibit the progression of intestinal fibrosis and improve clinical feasibility.

TGF-β1 is currently recognized as the most potent stimulating factor to activate fibroblasts *in vivo* and *in vitro*. To explore the mechanisms of the antifibrotic effects of pitavastatin, we investigated its roles in activation, proliferation, migration, inflammatory factor expression, and collagen synthesis of human colonic fibroblasts. In our study, treatment of CCD-18Co cells with different concentrations of pitavastatin, alone or after 24 h of stimulation with rhTGF-β1, showed that TGF-β1 stimulation activated CCD-18Co cells and significantly increased the expression levels of fibrosis-related proteins. Moreover, pitavastatin dose-dependently attenuated basal and TGF-β1-induced increases in α-SMA, COL1A1, MMP-3, IGF-1, and IGF-1R protein expression. These results suggest that pitavastatin exerts antifibrotic effects by inhibiting CCD-18Co cell activation and modulating collagen synthesis. Similarly, numerous studies have demonstrated that statins prevent renal, pulmonary, hepatic, cardiovascular, and cutaneous fibrosis by inhibiting fibroblast activation, quiescent precursor cell epithelial-mesenchymal transition or endothelial-mesenchymal transition, and by modulating myofibroblast activity (22,28-31).

In intestinal fibrosis, an imbalance of MMPs and their inhibitors (TIMPs) contributes to ECM remodeling. In healthy tissues, MMPs are typically present in inactive forms with low expression; however, their expression and activity are upregulated during inflammatory processes, leading to tissue damage (32). In the present study, MMP-3 and MMP-2 were shown to be upregulated in our DSS-induced colitis model, consistent with earlier studies (33,34). However, treatment with pitavastatin significantly reduced their expression levels. Notably, in contrast to pitavastatin's inhibition of MMP-3 expression, we found that pitavastatin dose-dependently enhanced the basal and TGF-β1-induced increase in MMP-9 protein expression in CCD-18Co cells. However, in recent preclinical antifibrotic studies, statin treatment had variable effects on MMP-9 expression. Qin et al. (35) reported that inhibition of cardiac fibrosis by simvastatin may be mediated by reduced levels of MMP-9 in mouse models. However, rosuvastatin treatment prevented progressive kidney inflammation and fibrosis in stroke-prone rats by preventing the decrease in MMP-9 activity (36). Fluvastatin was found to reduce the formation of intra-abdominal adhesions by promoting the expression of MMP-9 in an experimental rat model (37). These seemingly inconsistent findings may be due to different statin drugs having specific effects on different tissues, organs, and cells. Notably, however, the onset, progression, and reversal of fibrosis is a continuous and dynamic process, and the gene expression levels detected at a certain stage or point do not comprehensively represent changes in the rate of progression of the disease. During ECM synthesis and degradation, the MMP/TIMP ratio may better reflect the dynamic changes. Therefore, whether pitavastatin also enhances the expression of MMP-9 to exert antifibrotic effects *in vivo* requires further investigation, along with the changes in the MMP-9/TIMP ratio that occur during intestinal fibrosis.

We also demonstrated that pitavastatin suppressed the proliferation and migration of activated colonic fibroblasts. This is consistent with previously reported findings that statins inhibit the growth of cells in multiple-tissue fibrosis (38,39). Similar to previous findings, the present study confirmed that pitavastatin inhibited the expression of IGF-1 and IGF-1R in a dose-dependent manner (40). To further investigate whether the antifibrotic effects of pitavastatin act through IGF-1/IGF-1R, we co-treated CCD-18Co cells with IGF-1R-specific siRNA. The results confirmed that the upregulation of MMP-9 protein expression by pitavastatin was significantly inhibited when IGF-1/IGF-1R was silenced. This finding suggests that pitavastatin may exert its antifibrotic effect in CCD-18Co cells by upregulating MMP-9 expression through, or partly through, IGF-1/IGF-1R. However, we also found that silencing IGF-1/IGF-1R slightly reduced the basal and TGF-β1-induced increase in MMP-9 expression, indicating that IGF-1/IGF-1R may directly or indirectly regulate MMP-9. Nevertheless, the role of the *IGF-1* gene in the antifibrotic properties of statins and its underlying mechanisms require further study.

Several different cell types are involved in the process of intestinal fibrosis. This study was designed to investigate the effect of pitavastatin on fibroblast activation. Further research is required to confirm the effect of pitavastatin on other cellular phenotypic changes during fibrosis, including the epithelial-mesenchymal transition, endothelial-mesenchymal transition, and activation of immune cells. Additionally, although the results of our animal experiments were validated at the protein level, only immunohistochemical staining was employed. Further verification using protein quantification assays, such as western blot, is still needed. Furthermore, while our cellular experiments evaluated results at the protein level, mRNA analysis might provide further insights.

## Conclusion

We demonstrated that pitavastatin alleviated intestinal fibrosis and histological damage in mice with DSS-induced chronic colitis. Additionally, it inhibited collagen synthesis as well as the activation, proliferation, and migration of human colon fibroblasts. The underlying mechanisms may involve the promotion of MMP-9 expression through the IGF-1/IGF-1R pathway. These findings indicated that pitavastatin may be a promising candidate for further investigation in the treatment of intestinal fibrosis and provide additional evidence supporting the antifibrotic effects of statins.

## Supplementary Material

Click to view [pdf].
