# Supplementary material for: Pitavastatin reduces intestinal fibrosis in chronic colitis and inhibits colon fibroblast activation by enhancing MMP-9 expression via the IGF-1/IGF-1R pathway
Source: Braz J Med Biol Res. 2025 Aug 22;58:e14540. doi: 10.1590/1414-431X2025e14540 (PMC12377707; doi:10.1590/1414-431X2025e14540)
Supplement: Supplementary file 1 [file 1414-431X-bjmbr-58-e14540-suppl.pdf]

Table S1. Disease activity index (DAI) scoring system of dextran sodium sulfate (DSS)-induced colitis.

Score	Weight loss (%)	Stool consistency	Blood in feces
0	No weight loss	Normal	Normal
1	1-5		
2	>5-10	Loose stool	Positive fecal occult blood
3	>10-15		
4	>15	Pasty or thin water stool	Visible blood in feces

Calculation formula: DAI = (Weight Loss Score + Stool Consistency Score + Blood in Feces Score) / 3.
